# Fast smooth second-order sliding mode control for systems with additive colored noises

**DOI:** 10.1371/journal.pone.0178455

**Published:** 2017-05-31

**Authors:** Pengfei Yang, Yangwang Fang, Youli Wu, Yunxia Liu, Danxu Zhang

**Affiliations:** 1 School of Aeronautics and Astronautics Engineering, Air Force Engineering University, Xi’an, Shaanxi, China; 2 College of Education, Hunan University of Science and Technology, Xiangtan, Hunan, China; Tongji University, CHINA

## Abstract

In this paper, a fast smooth second-order sliding mode control is presented for a class of stochastic systems with enumerable Ornstein-Uhlenbeck colored noises. The finite-time mean-square practical stability and finite-time mean-square practical reachability are first introduced. Instead of treating the noise as bounded disturbance, the stochastic control techniques are incorporated into the design of the controller. The finite-time convergence of the prescribed sliding variable dynamics system is proved by using stochastic Lyapunov-like techniques. Then the proposed sliding mode controller is applied to a second-order nonlinear stochastic system. Simulation results are presented comparing with smooth second-order sliding mode control to validate the analysis.

## Introduction

Sliding mode control (SMC) is well known for its robustness to system parameter variations and external disturbances[1,2]. SMC has extensive applications in practice, such as robots, aircrafts, DC and AC motors, power systems, process control and so on. Recently, using SMC strategy to the nonlinear stochastic systems modeled by the Itô stochastic differential equations with multiplicative noise has been gaining much investigation, see [3–6] and references therein.

The existing research findings applying SMC to the stochastic systems always treat the stochastic noise as bounded uncertainties. These methods need to know the upper bound of the noise and they are comparatively more conservative control strategy, which ensure the robustness at the cost of losing control accuracy. Some literatures derived SMC for the stochastic systems described in Itô’s form applying stability in probability[3], which was proved to be unstable under the second moment stability concept[7]. By comparison, mean-square stability is more practical for engineering application. Wu et al.[8] designed SMC guaranteeing the mean-square exponential stability for the continuous-time switched stochastic systems with multiplicative noise. However, the control signal in [8] switches frequently and the results cannot be extended to stochastic systems with additive noise.

One disadvantage of classical SMC is that the sliding variable cannot converge to the sliding surface in finite time. Finite-time convergence has been widely investigated in the control systems. Shang discussed the finite-time state consensus problems for multi-agent systems[9,10], and further investigated the finite-time cluster average consensus in bidirectional networks and the fixed-time group consensus problem for a leader-follower network[11,12]. It is urgent to deduce finite-time convergence sliding mode method for stochastic systems.

In addition, traditional SMC has restrictions such as the relative degree constraint and the high frequency control switching that may easily cause chattering effect[13]. Rahmani designed an adaptive neural network to approximate the system uncertainties and unknown disturbances to reduce chattering phenomena, and proposed controllers combining adaptive neural network with sliding mode control methods[14,15]. Ref.[16] designed a fractional order PID controller to a bio-inspired robot manipulator using bat algorithm. Higher-order sliding mode control (HOSM) also mitigates the problems associated with SMC[17–21]. In the past decades, HOSM has found a variety of application in the robust control of uncertain systems[22,23]. But HOSM for the stochastic systems is remaining poorly investigated.

Aiming at the defects of the above mentioned research, a smooth control law for a class of nonlinear stochastic systems with Ornstein-Uhlenbeck colored noise is developed in this paper. By using stochastic Lyapunov-like techniques, a sufficient condition of finite-time convergence is derived under the mean-square practical stability concept. Finally, some experimental results are presented to validate the proposed controller.

## Materials and methods

### Problem statement

Let *α* > 0 and *σ* = *const*., the following Itô stochastic differential equation

$$\dot{\eta }(t)=-\alpha \eta (t)+\sigma \zeta (t),\;\eta (t_{0})=0$$ (1)

is called Langevin equation, where *ζ*(*t*) is a standard scalar Gaussian white noise. The solution *η*(*t*) (*t* ≥ 0) is called Ornstein-Uhlenbeck process, which is a colored noise[24].

Consider single-input single-output (SISO) dynamics with denumerable Ornstein-Uhlenbeck colored noises

$$\dot{s}=f(t)+g(t)u+d(t)+\sum_{i=1}^{l}h_{i}{\bar{\eta }}_{i}$$ (2)

where *h*_*i*_ are constants; *f*(*t*), *g*(*t*) are given sufficiently smooth function and *g*(*x*) ≠ 0; *d*(*t*) presents unmodeled dynamics, parametric uncertainties and external disturbances, which is assumed to be sufficiently smooth; ${\bar{\eta }}_{i}$ are mutually independent Ornstein-Uhlenbeck colored noises with parameters *α*_*i*_ and ${\bar{\sigma }}_{i}$. *s* can be interpreted as dynamics of the sliding variable *s* ∈ ***R***^1^ calculated along the system trajectory and *s* = 0 expresses sliding manifold; *u* ∈ ***R***^1^ is the control input. In order to prevent the chattering and exploit the benefits of a sliding mode controller in a real-life system, a smooth control, which can provide a finite time convergence $s,\dot{s}\to 0$, is urgently needed.

### Stochastic fast smooth second-order sliding mode control

#### Problem formulation and definitions

Obviously, system (2) is a stochastic nonlinear system with additive noise, meaning that the system does not have any equilibrium point. This system is unstable under the concept of stability in the sense of Lyapunov, but may also exhibit interesting behavior similar to a conventional stable system near equilibrium[25,26]. That is to say, the desired state is mathematically unstable, but the system may oscillate sufficiently near this state so that the performance is considered acceptable[27]. Motivated by this fact, practical stability is proposed by LaSalle and Lefschetz[28] and was developed by Martynyuk, Lakshmikantham and Leela et al[29,30].

As a natural extension of the traditional concepts of practical stability, mean-square stability, and finite-time reachability, we shall introduce the concepts of finite-time mean-square practical stability and finite-time mean-square practical reachability. These concepts are concerned with bringing the system trajectory into a bounded neighborhood of a given point or manifold.

Consider the following stochastic dynamical system

$$\dot{x}(t)=f(t,x)+h(t,x)\eta$$ (3)

where ***f*** ∈ *C*[*R*^+^ × *R*^*n*^,*R*^*n*^], ***h*** ∈ *C*[*R*^+^ × *R*^*n*^,*R*^*n*^]; *η* is 1-dimensional stochastic process. Denote *x*(*t*) = *x*(*t*,*t*_0_,*x*_0_) as the solution of (3) under the initial condition (*t*_0_,***x***_0_). Let ***s*** = ***s***(*t*,***x***) = 0 be the chosen sliding manifold of the system.

**Definition 1 (FTMSP):** The solution *x*(*t*) of system (3) is said to be

(S_1_) mean-square practically stable with respect to (*λ*,*A)*, if given a pair of positive numbers (*λ*,*A*) with *A* > *λ* > 0 such that ‖***x***_0_‖ < *λ* implies E‖***x***‖^2^ < *A*,*t* ≥ *t*_0_ for some *t*_0_ ∈ *R*^+^;

(S_2_) mean-square uniformly practically stable with respect to (*λ*,*A*), if (S_1_) holds for all *t*_0_ ∈*R*^+^;

(S_3_) finite-time mean-square practically stable with respect to (λ,ε), if for every *ε*, there exist *T* and *λ* such that ‖***x***_0_‖ ≤ *λ* implies E‖***x***‖^2^ < *ε*,*t* ≥ *t*_0_ + *T* for some *t*_0_ ∈*R*^+^;

(S_4_) finite-time mean-square uniformly practically stable with respect to (*λ*,*ε*), if (S_3_) holds for all *t*_0_ ∈*R*^+^;

(S_5_) finite-time mean-square strongly practically stable with respect to (*λ*,*ε*), if (S_1_) and (S_3_) hold simultaneously;

(S_6_) finite-time mean-square strongly uniformly practically stable with respect to (*λ*,*ε*), if (S_2_) and (S_4_) hold simultaneously.

**Remark 1**: Unlike definitions in [28,29], which emphasize the boundedness of the system trajectory, the definition we taken here focus far more on the convergence of the system trajectory.

**Definition 2 (FTMSR):** The sliding manifold ***s***=0 is said to be

(R_1_) finite-time mean-square practically reached, if given a pair of positive numbers (*λ*,*ε*), *λ* = *λ*_1_ + *λ*_2_ and *ε* = *ε*_1_ + *ε*_2_, there exists a finite setting time *T* = *T*(*t*_0_,*ε*), such that

$$\left\{ \begin{array}{l} {\left\|s(x_{0},t_{0})\right\|}^{2}\leq \lambda_{1}\\ {\left\|\dot{s}(x_{0},t_{0})\right\|}^{2}\leq \lambda_{2} \end{array}\right.$$

implies E‖***s***(***x*,***t*)***‖***^2^ ≤ *ε*,∀*t > t*_0_ *+ T* for some *t*_0_ ∈*R*^+^;

(R_2_) finite-time mean-square uniformly practically reached, if (R_1_) holds for all *t*_0_ ∈*R*^+^;

(R_3_) second-order finite-time mean-square practically reached, if given a pair of positive numbers (*λ*,*ε*), *λ* = *λ*_1_ + *λ*_2_ and *ε* = *ε*_1_ + *ε*_2_, there exists a finite setting time *T* = *T*(*t*_0_,*ε*), such that

$$\left\{ \begin{array}{l} {\left\|s(x_{0},t_{0})\right\|}^{2}\leq \lambda_{1}\\ {\left\|\dot{s}(x_{0},t_{0})\right\|}^{2}\leq \lambda_{2} \end{array}\right.$$

implies

$$\left\{ \begin{array}{l} E{\left\|s(x,t)\right\|}^{2}\leq \varepsilon_{1}\\ E{\left\|\dot{s}(x,t)\right\|}^{2}\leq \varepsilon_{2} \end{array}\right.,\forall t>t_{0}+T$$

for some *t*_0_ ∈*R*^+^;

(R_4_) second-order finite-time mean-square uniformly practically reached, if (R_3_) holds for all *t*_0_ ∈*R*^+^;

#### Stochastic fast smooth second-order sliding mode control

Consider system Eq (2), denote $\eta_{i}=h_{i}{\bar{\eta }}_{i}$, $\sigma_{i}={\bar{\sigma }}_{i}h_{i}$ and we have

$${\dot{\eta }}_{i}=h_{i}{\dot{\bar{\eta }}}_{i}=h_{i}\left[-\alpha_{i}{\bar{\eta }}_{i}(t)+{\bar{\sigma }}_{i}\zeta_{i}(t)\right]=-\alpha_{i}\eta_{i}(t)+\sigma_{i}\zeta_{i}(t)$$ (4)

meaning that *η*_*i*_ is a Ornstein-Uhlenbeck noise with parameters *α*_*i*_ and *σ*_*i*_, so the coefficient *h*_*i*_ can be merged by substitute *η*_*i*_ into (2) to get

$$\dot{s}=f(t)+g(t)u+d(t)+\sum_{i=1}^{l}\eta_{i}$$ (5)

Consider system Eq (5), the dynamics of the sliding variable is designed as the following form:

$$\left\{ \begin{array}{l} {\dot{\mu }}_{1}=-k_{1}{\left|\mu_{1}\right|}^{\frac{m-1}{m}}\mathrm{sgn}\left(\mu_{1}\right)-k_{2}\mu_{1}-k_{3}\left|\mu_{2}\right|\mathrm{sgn}(\mu_{1})+\sum_{i=1}^{l}\eta_{i}\\ {\dot{\mu }}_{2}=-k_{4}{\left|\mu_{1}\right|}^{\frac{m-2}{m}}\mathrm{sgn}\left(\mu_{2}\right)-k_{5}\mu_{2} \end{array}\right.$$ (6)

where *μ*_1_ = *s*; *m* and *k*_*i*_ are positive constants and *m* > 2; *η*_*i*_ are Ornstein-Uhlenbeck colored noises expressed in (4).

Let ***μ*** = [*μ*_1_, *μ*_2_, *η*_1_, *η*_2_, ⋯, *η*_1_]^*T*^, the following Itô stochastic differential equation can be got by combining (5) and (6) together:

$$\left[\begin{array}{c} {\dot{\mu }}_{1}\\ {\dot{\mu }}_{2}\\ {\dot{\eta }}_{1}\\ \vdots \\ {\dot{\eta }}_{l} \end{array}\right]=\left[\begin{array}{c} -k_{1}{\left|\mu_{1}\right|}^{\frac{m-1}{m}}\mathrm{sgn}\left(\mu_{1}\right)-k_{2}\mu_{1}-k_{3}\left|\mu_{2}\right|\mathrm{sgn}(\mu_{1})+\sum_{i=1}^{l}\eta_{i}\\ -k_{4}{\left|\mu_{1}\right|}^{\frac{m-2}{m}}\mathrm{sgn}\left(\mu_{2}\right)-k_{5}\mu_{2}\\ -\alpha_{1}\eta_{1}\\ \vdots \\ -\alpha_{l}\eta_{l} \end{array}\right]+\left[\begin{array}{c} 0\\ 0\\ \sigma_{1}\\ \vdots \\ \sigma_{l} \end{array}\right]\zeta$$ (7)

then a stochastic system with respect to the state vector ***μ*** can be represented as

dμ=f(μ)dt+gdW(t) (8)

where

$$\begin{array}{l} f\left(\mu \right)=\left[\begin{array}{c} -k_{1}{\left|\mu_{1}\right|}^{\frac{m-1}{m}}\mathrm{sgn}\left(\mu_{1}\right)-k_{2}\mu_{1}-k_{3}\left|\mu_{2}\right|\mathrm{sgn}(\mu_{1})+\sum_{i=1}^{l}\eta_{i}\\ -k_{4}{\left|\mu_{1}\right|}^{\frac{m-2}{m}}\mathrm{sgn}\left(\mu_{2}\right)-k_{5}\mu_{2}\\ -\alpha_{1}\eta_{1}\\ \vdots \\ -\alpha_{l}\eta_{l} \end{array}\right]\\ g={\left[0\;0\;\sigma_{1}\;\cdots \;\sigma_{l}\right]}^{T} \end{array}$$ (9)

Let the sliding variable dynamics be of the form (6) and in accordance with the sliding variable system (5), the SFS-SOSM controller is selected as

$$\left\{ \begin{array}{l} \;u=g^{-1}(t)[-k_{1}{\left|\mu_{1}\right|}^{\frac{m-1}{m}}\mathrm{sgn}\left(\mu_{1}\right)-k_{2}\mu_{1}-k_{3}\left|\mu_{2}\right|\mathrm{sgn}(\mu_{1})-f(t)-\hat{d}(t)]\\ {\dot{\mu }}_{2}=-k_{4}{\left|\mu_{1}\right|}^{\frac{m-2}{m}}\mathrm{sgn}\left(\mu_{2}\right)-k_{5}\mu_{2} \end{array}\right.$$ (10)

where $\hat{d}(t)$ is the estimation of uncertain function by means of high-order sliding-mode observer presented in [22].

Hereafter, FTMSP and FTMSR are employed to analyze the reachability of the sliding manifold.

#### Finite time convergence analysis

Based on the definition proposed above, we give the following theorem:

**Theorem 1**: Consider the stochastic nonlinear system (6) with respect to the sliding variable *s*, let

$$P_{i}=\frac{k_{5}}{\alpha_{i}(\alpha_{i}+k_{2})}+\frac{k_{2}k_{5}}{\alpha_{i}},\;Q_{i}=\frac{k_{5}}{\alpha_{i}+k_{2}},\;i=1,2,\cdots ,l$$ (11)

where *m* > 2, *α*_*i*_ > 0 (*i* = 1,2,⋯,*l*), *k*_*j*_ > 0 (*j* = 1,⋯,5). Constructing the following matrix

$$\Lambda =\frac{1}{2}{\left[\begin{array}{cccccc} k_{5} & 0 & Q_{1} & Q_{2} & \cdots & Q_{l}\\ 0 & k_{2} & 0 & 0 & \cdots & 0\\ Q_{1} & 0 & P_{1} & 0 & \cdots & 0\\ Q_{2} & 0 & 0 & P_{2} & \cdots & 0\\ \vdots & \vdots & \vdots & \vdots & \ddots & \\ Q_{l} & 0 & 0 & 0 & \cdots & P_{l} \end{array}\right]}_{\left(l+2)(l+2\right)}$$ (12)

and assuming that

(i) $\varepsilon =[1+{\left(k_{1}+k_{2}+k_{3}+l\right)}^{2}]\bar{\varepsilon }$ and the following inequality holds

$$\bar{\varepsilon }\geq \frac{\gamma_{2}}{\lambda_{\min}(\Lambda )\gamma_{1}}$$ (13)

where

$$\gamma_{1}=\frac{k_{2}k_{5}}{\lambda_{\max}(\Lambda )},\;\gamma_{2}=\frac{1}{2}\sum_{i=1,j=1,i\neq j}^{l}\left[\sqrt{\frac{1}{\alpha_{i}\alpha_{j}}}(Q_{i}+Q_{j})\sigma_{i}\sigma_{j}\right]+\frac{1}{2}\sum_{i=1}^{l}P_{i}\sigma_{i}^{2}$$

*σ*_*i*_ are the parameter of the colored noise mentioned in (4).

(ii) Positive number *λ* satisfies

$$\lambda >\frac{\gamma_{2}}{\lambda_{\max}(\Lambda )\gamma_{1}}-\sum_{i=1}^{l}\frac{\sigma_{i}^{2}}{2\alpha_{i}}$$ (14)

Then the prescribed sliding variable dynamics system (6) is finite-time mean-square practically stable, and the proposed control (10) is an SFS-SOSM control. The sliding manifold *s* = 0 can be second-order mean-square practically reached in finite time.

**Proof:** According to the definition given before, we want to prove that for the prescribed sliding variable dynamics system (6), if given positive numbers (*λ*,*ε*), *λ* = *λ*_1_ + *λ*_2_ and *ε* = *ε*_1_+ *ε*_2_, there exists a finite setting time *T* = *T*(*t*_0_,*ε*), such that

$$\left\{ \begin{array}{l} {\left|s(x_{0},t_{0})\right|}^{2}\leq \lambda_{1}\\ {\left|\dot{s}(x_{0},t_{0})\right|}^{2}\leq \lambda_{2} \end{array}\right.$$

implies

$$\left\{ \begin{array}{l} E{\left|s(x,t)\right|}^{2}\leq \varepsilon_{1}\\ E{\left|\dot{s}(x,t)\right|}^{2}\leq \varepsilon_{2} \end{array}\right.,\forall t>t_{0}+T$$

To prove this, aiming at the augmented system (8), we define the Lyapunov-like functional as

$$V=\frac{1}{2}k_{5}\mu_{1}^{2}+\frac{1}{2}k_{2}\mu_{2}^{2}+\frac{1}{2}\sum_{i=1}^{l}\left[\frac{k_{5}}{\alpha_{i}(\alpha_{i}+k_{2})}+\frac{k_{2}k_{5}}{\alpha_{i}}\right]\eta_{i}^{2}+\sum_{i=1}^{l}\frac{k_{5}}{\alpha_{i}+k_{2}}\left|\mu_{1}\right|\left|\eta_{i}\right|$$ (15)

Since *V*(***μ***) is continuous but not differentiable, a nonsmooth version of Lyapunov’s theory is required, which shows that one can just consider the points where *V*(***μ***) is differentiable[28,29]. This argument is valid in all the proofs of this paper.

The substitution ξ = [|μ_1_|,|μ_2_|,|η_1_|,|η_2_|,⋯,|η_1_|]^T^ brings the proposed functional (15) to a quadratic form

$$V=\xi^{T}\Lambda \xi$$ (16)

where ***Λ*** is given in (12). It is obvious that ***Λ*** is positive definite since *α*_*i*_ > 0 (*i* = 1,2,⋯,*l*), *k*_*j*_ > 0 (*j* = 1,⋯,5). Note that *V*(***μ***) is positive definite and unbounded, the following inequalities can be obtained based on Rayleigh-Ritz Theorem

$$\lambda_{\min}(\Lambda )E({\left\|\xi \right\|}^{2})\leq EV\leq \lambda_{\max}(\Lambda )E({\left\|\xi \right\|}^{2})$$ (17)

where ${\left\|\xi \right\|}^{2}=\mu_{1}^{2}+\mu_{2}^{2}+\eta_{1}^{2}+\eta_{2}^{2}+\cdots +\eta_{l}^{2}$ is the Euclidean norm of *ξ*, *λ*_min_(***Λ***) and *λ*_max_(***Λ***) are minimal and maximal eigenvalues of ***Λ***.

We denote the infinitesimal generator by *ℒ*. Appling infinitesimal generator along with system (8), we have

$$ℒV=\left[\begin{array}{ccccc} \frac{\partial V}{\partial \mu_{1}} & \frac{\partial V}{\partial \mu_{2}} & \frac{\partial V}{\partial \eta_{1}} & \cdots & \frac{\partial V}{\partial \eta_{l}} \end{array}\right]\left[\begin{array}{c} {\dot{\mu }}_{1}\\ {\dot{\mu }}_{2}\\ {\dot{\eta }}_{1}\\ \vdots \\ {\dot{\eta }}_{l} \end{array}\right]+\frac{1}{2}trace(g^{T}\Delta Vg)$$ (18)

Let

$$ℒV_{1}=\left[\begin{array}{ccccc} \frac{\partial V}{\partial \mu_{1}} & \frac{\partial V}{\partial \mu_{2}} & \frac{\partial V}{\partial \eta_{1}} & \cdots & \frac{\partial V}{\partial \eta_{l}} \end{array}\right]\left[\begin{array}{c} {\dot{\mu }}_{1}\\ {\dot{\mu }}_{2}\\ {\dot{\eta }}_{1}\\ \vdots \\ {\dot{\eta }}_{l} \end{array}\right],\;ℒV_{2}=\frac{1}{2}trace(g^{T}\Delta Vg)$$

*ℒV*_1_ can be expanded and the following inequality holds

$$\begin{array}{l} ℒV_{1}={\left[\begin{array}{c} k_{5}\mu_{1}+\mathrm{sgn}(\mu_{1})\sum_{i=1}^{l}Q_{i}\left|\eta_{i}\right|\\ k_{2}\mu_{2}\\ P_{1}\eta_{1}+Q_{1}\left|\mu_{1}\right|\mathrm{sgn}(\eta_{1})\\ \vdots \\ P_{l}\eta_{l}+Q_{l}\left|\mu_{1}\right|\mathrm{sgn}(\eta_{l}) \end{array}\right]}^{T}\cdot \left[\begin{array}{c} -k_{1}{\left|\mu_{1}\right|}^{\frac{m-1}{m}}\mathrm{sgn}\left(\mu_{1}\right)-k_{2}\mu_{1}-k_{3}\left|\mu_{2}\right|\mathrm{sgn}(\mu_{1})+\sum_{i=1}^{l}\eta_{i}\\ -k_{4}{\left|\mu_{1}\right|}^{\frac{m-2}{m}}\mathrm{sgn}\left(\mu_{2}\right)-k_{5}\mu_{2}\\ -\alpha_{1}\eta_{1}\\ \vdots \\ -\alpha_{l}\eta_{l} \end{array}\right]\\ \;=-k_{1}k_{5}{\left|\mu_{1}\right|}^{\frac{2m-1}{m}}-k_{2}k_{5}\mu_{1}^{2}-k_{3}k_{5}\left|\mu_{1}\right|\left|\mu_{2}\right|+k_{5}\mu_{1}\sum_{i=1}^{l}\eta_{i}\\ \;+(\sum_{i=1}^{l}Q_{i}\left|\eta_{i}\right|)\cdot \left[-k_{1}{\left|\mu_{1}\right|}^{\frac{m-1}{m}}-k_{3}\left|\mu_{2}\right|\right]-k_{2}\left|\mu_{1}\right|\sum_{i=1}^{l}Q_{i}\left|\eta_{i}\right|+\mathrm{sgn}(\mu_{1})\sum_{i=1}^{l}Q_{i}\left|\eta_{i}\right|\cdot \sum_{i=1}^{l}\eta_{i}\\ \;-k_{2}k_{4}{\left|\mu_{1}\right|}^{\frac{2m-2}{m}}-k_{2}k_{5}\mu_{2}^{2}-P_{1}\alpha_{1}\eta_{1}^{2}-Q_{1}\alpha_{1}\left|\mu_{1}\right|\left|\eta_{1}\right|-\cdots -P_{l}\alpha_{l}\eta_{l}^{2}-Q_{l}\alpha_{l}\left|\mu_{1}\right|\left|\eta_{l}\right|\\ \;\leq -k_{2}k_{5}\mu_{1}^{2}-k_{2}k_{5}\mu_{2}^{2}-\sum_{i=1}^{l}\alpha_{i}P_{i}\eta_{i}^{2}+\sum_{i=1}^{l}(k_{5}-k_{2}Q_{i}-\alpha_{i}Q_{i})\left|\mu_{1}\right|\left|\eta_{i}\right|\\ \;+(Q_{1}\left|\eta_{1}\right|+Q_{2}\left|\eta_{2}\right|+\cdots +Q_{l}\left|\eta_{l}\right|)(\left|\eta_{1}\right|+\left|\eta_{2}\right|+\cdots +\left|\eta_{l}\right|) \end{array}$$ (19)

Notice that

$$k_{5}-k_{2}Q_{i}-\alpha_{i}Q_{i}=k_{5}-(\alpha_{i}+k_{2})\frac{k_{5}}{\alpha_{i}+k_{2}}=0,\;i=1,2,\cdots ,l$$ (20)

Then the following inequality can be deduced

$$\begin{array}{c} ℒV_{1}\leq -k_{2}k_{5}\mu_{1}^{2}-k_{2}k_{5}\mu_{2}^{2}-\sum_{i=1}^{l}\alpha_{i}P_{i}\eta_{i}^{2}+\sum_{i=1}^{l}Q_{i}\eta_{i}^{2}+\sum_{i=1,j=1,i\neq j}^{l}(Q_{i}+Q_{j})\left|\eta_{i}\right|\left|\eta_{j}\right|\\ =-k_{2}k_{5}\mu_{1}^{2}-k_{2}k_{5}\mu_{2}^{2}-\sum_{i=1}^{l}(\alpha_{i}P_{i}-Q_{i})\eta_{i}^{2}+\sum_{i=1,j=1,i\neq j}^{l}(Q_{i}+Q_{j})\left|\eta_{i}\right|\left|\eta_{j}\right| \end{array}$$ (21)

Furthermore,

$$\alpha_{i}P_{i}-Q_{i}=\alpha_{i}\left[\frac{k_{5}}{\alpha_{i}(\alpha_{i}+k_{2})}+\frac{k_{2}k_{5}}{\alpha_{i}}\right]-\frac{k_{5}}{\alpha_{i}+k_{2}}=k_{2}k_{5},\;i=1,2,\cdots ,l$$ (22)

then we have

$$\begin{array}{l} ℒV_{1}\leq -k_{2}k_{5}\mu_{1}^{2}-k_{2}k_{5}\mu_{2}^{2}-\sum_{i=1}^{l}k_{2}k_{5}\eta_{i}^{2}+\sum_{i=1,j=1,i\neq j}^{l}(Q_{i}+Q_{j})\left|\eta_{i}\right|\left|\eta_{j}\right|\\ \;=-k_{2}k_{5}{\left\|\xi \right\|}^{2}+\sum_{i=1,j=1,i\neq j}^{l}(Q_{i}+Q_{j})\left|\eta_{i}\right|\left|\eta_{j}\right| \end{array}$$ (23)

The inequality about *ℒV*_2_ can be deduced according to the properties of the matrix trace as

$$\begin{array}{l} ℒV_{2}=\frac{1}{2}trace(g^{T}\Delta Vg)=\frac{1}{2}g^{T}\Delta Vg\\ \;=\frac{1}{2}\left[\begin{array}{ccccc} 0 & 0 & \sigma_{1} & \cdots & \sigma_{l} \end{array}\right]\left[\begin{array}{ccccc} \frac{\partial^{2}V}{\partial \mu_{1}^{2}} & & & & \\ & \frac{\partial^{2}V}{\partial \mu_{2}^{2}} & & & \\ & & \frac{\partial^{2}V}{\partial \eta_{1}^{2}} & & \\ & & & \ddots & \\ & & & & \frac{\partial^{2}V}{\partial \eta_{l}^{2}} \end{array}\right]\left[\begin{array}{c} 0\\ 0\\ \sigma_{1}\\ \vdots \\ \sigma_{l} \end{array}\right]\\ \;=\frac{1}{2}\sum_{i=1}^{l}\left(\sigma_{i}^{2}\frac{\partial^{2}V}{\partial \eta_{i}^{2}}\right)=\frac{1}{2}\sum_{i=1}^{l}P_{i}\sigma_{i}^{2} \end{array}$$ (24)

Substitute(23), (24) into (18) to get

$$ℒV\leq -k_{2}k_{5}{\left\|\xi \right\|}^{2}+\sum_{i=1,j=1,i\neq j}^{l}(Q_{i}+Q_{j})\left|\eta_{i}\right|\left|\eta_{j}\right|+\frac{1}{2}\sum_{i=1}^{l}P_{i}\sigma_{i}^{2}$$ (25)

According to Itô’s formula, it follows that

$$\begin{array}{l} \left(EV{)}^{\prime }=E(LV\right)\\ \;=-k_{2}k_{5}E({\left\|\xi \right\|}^{2})+\sum_{i=1,j=1,i\neq j}^{l}(Q_{i}+Q_{j})E\left|\eta_{i}\right|E\left|\eta_{j}\right|+\frac{1}{2}\sum_{i=1}^{l}P_{i}\sigma_{i}^{2} \end{array}$$ (26)

Since *η*_*i*_ are mutually independent, utilizing E[*η*^2^ (*t*)] ≤ *σ*^2^/2*α* and Rao inequality[31] to obtain:

$${\left(E\left|\eta_{i}(t)\right|\right)}^{2}\leq E1E({\left|\eta_{i}(t)\right|}^{2})=E[\eta_{i}^{2}(t)]\leq \frac{\sigma_{i}^{2}}{2\alpha_{i}}$$ (27)

Then inequality (26) can be further represented as

$$\begin{array}{l} (EV{)}^{\prime }\leq -k_{2}k_{5}E({\left\|\xi \right\|}^{2})+\sum_{i=1,j=1,i\neq j}^{l}(Q_{i}+Q_{j})\sqrt{\frac{\sigma_{i}^{2}}{2\alpha_{i}}}\sqrt{\frac{\sigma_{j}^{2}}{2\alpha_{j}}}+\frac{1}{2}\sum_{i=1}^{l}P_{i}\sigma_{i}^{2}\\ \;\leq -\frac{k_{2}k_{5}}{\lambda_{\max}(\Lambda )}EV+\frac{1}{2}\sum_{i=1,j=1,i\neq j}^{l}\left[\sqrt{\frac{1}{\alpha_{i}\alpha_{j}}}(Q_{i}+Q_{j})\sigma_{i}\sigma_{j}\right]+\frac{1}{2}\sum_{i=1}^{l}P_{i}\sigma_{i}^{2}\\ \;=-\gamma_{1}EV+\gamma_{2} \end{array}$$ (28)

where

$$\gamma_{1}=\frac{k_{2}k_{5}}{\lambda_{\max}(\Lambda )},\;\gamma_{2}=\frac{1}{2}\sum_{i=1,j=1,i\neq j}^{l}\left[\sqrt{\frac{1}{\alpha_{i}\alpha_{j}}}(Q_{i}+Q_{j})\sigma_{i}\sigma_{j}\right]+\frac{1}{2}\sum_{i=1}^{l}P_{i}\sigma_{i}^{2}$$ (29)

It is obvious that *γ*_1_,*γ*_2_ > 0.

Since the solution of the differential equation

$$\dot{\varphi }=-\gamma_{1}\varphi +\gamma_{2},\;\varphi (t_{0})=\varphi_{0}\geq 0$$ (30)

is given by

$$\varphi (t)=(\varphi_{0}-\frac{\gamma_{2}}{\gamma_{1}})e^{-\gamma_{1}(t-t_{0})}+\frac{\gamma_{2}}{\gamma_{1}}$$ (31)

it follows from the comparison principle[32] that E*V*(*t*) ≤ *φ*(*t*) when E*V(t*_0_*) ≤ φ*_0_. From (31) we can claim that the following inequality holds.

$$EV(t)\leq (EV(t_{0})-\frac{\gamma_{2}}{\gamma_{1}})e^{-\gamma_{1}(t-t_{0})}+\frac{\gamma_{2}}{\gamma_{1}}$$ (32)

From the initial conditions, we have ${\left|s(x_{0},t_{0})\right|}^{2}+{\left|\dot{s}(x_{0},t_{0})\right|}^{2}\leq \lambda$. So the initial condition of the constructed vector *ξ* can be got as

$$E{\left\|\xi (x_{0},t_{0})\right\|}^{2}=E{\left|\mu_{1}(x_{0},t_{0})\right|}^{2}+E{\left|\mu_{2}(x_{0},t_{0})\right|}^{2}+\sum_{i=1}^{l}E\eta_{i}^{2}<\lambda +\sum_{i=1}^{l}E\eta_{i}^{2}\leq \lambda +\sum_{i=1}^{l}\frac{\sigma_{i}^{2}}{2\alpha_{i}}$$ (33)

For convenient, we denote

$$\xi_{0}=\xi (x_{0},t_{0}),\;\delta =\sum_{i=1}^{l}\frac{\sigma_{i}^{2}}{2\alpha_{i}}$$

and synthesize the results we have got in (17), (32), (33), the following inequality can be deduced

$$\begin{array}{l} E{\left\|\xi (t)\right\|}^{2}\leq \frac{EV(t)}{\lambda_{\min}(\Lambda )}\leq \frac{1}{\lambda_{\min}(\Lambda )}\{[EV_{0}-\frac{\gamma_{2}}{\gamma_{1}}]e^{-\gamma_{1}(t-t_{0})}+\frac{\gamma_{2}}{\gamma_{1}}\}\\ \;\leq \frac{1}{\lambda_{\min}(\Lambda )}\{[\lambda_{\max}(\Lambda )E{\left\|\xi_{0}\right\|}^{2}-\frac{\gamma_{2}}{\gamma_{1}}]e^{-\gamma_{1}(t-t_{0})}+\frac{\gamma_{2}}{\gamma_{1}}\}\\ \;<[\frac{\lambda_{\max}(\Lambda )}{\lambda_{\min}(\Lambda )}(\lambda +\delta )-\frac{\gamma_{2}}{\lambda_{\min}(\Lambda )\gamma_{1}}]e^{-\gamma_{1}(t-t_{0})}+\frac{\gamma_{2}}{\lambda_{\min}(\Lambda )\gamma_{1}} \end{array}$$ (34)

Let

$$H(t)=[\frac{\lambda_{\max}(\Lambda )}{\lambda_{\min}(\Lambda )}(\lambda +\delta )-\frac{\gamma_{2}}{\lambda_{\min}(\Lambda )\gamma_{1}}]e^{-\gamma_{1}(t-t_{0})}+\frac{\gamma_{2}}{\lambda_{\min}(\Lambda )\gamma_{1}}$$ (35)

Taking the derivative of *H*(*t*) and using condition (ii), the following inequality holds

$$\dot{H}(t)=-\gamma_{1}\left[\frac{\lambda_{\max}(\Lambda )}{\lambda_{\min}(\Lambda )}(\lambda +\delta )-\frac{\gamma_{2}}{\lambda_{\min}(\Lambda )\gamma_{1}}\right]e^{-\gamma_{1}(t-t_{0})}<0$$ (36)

meaning that *H*(*t*) is monotonically decreasing with respect to time *t*, and the theoretical lower bound *H*_min_ is

$$H_{\min}=\underset{t\to \infty }{\lim}H(t)=\frac{\gamma_{2}}{\lambda_{\min}(\Lambda )\gamma_{1}}$$ (37)

By condition (ii), we have $\bar{\varepsilon }\geq H_{\min}$, define *T* as the time taken to achieve $E{\left\|\xi (t)\right\|}^{2}=\bar{\varepsilon }$, then the expression of *T* can be deduced by

$$[\frac{\lambda_{\max}(\Lambda )}{\lambda_{\min}(\Lambda )}(\lambda +\delta )-\frac{\gamma_{2}}{\lambda_{\min}(\Lambda )\gamma_{1}}]e^{-\gamma_{1}T}+\frac{\gamma_{2}}{\lambda_{\min}(\Lambda )\gamma_{1}}=\bar{\varepsilon }$$ (38)

Solving (38) results in

$$T=\frac{1}{\gamma_{1}}\ln\left[\frac{\frac{\lambda_{\max}(\Lambda )}{\lambda_{\min}(\Lambda )}(\lambda +\delta )-\frac{\gamma_{2}}{\lambda_{\min}(\Lambda )\gamma_{1}}}{\bar{\varepsilon }-\frac{\gamma_{2}}{\lambda_{\min}(\Lambda )\gamma_{1}}}\right]$$ (39)

By Definition 1, we can claim that the augmented system (8) is finite-time mean-square uniformly practically stable with respect to $\left(\lambda ,\bar{\varepsilon }\right)$. Now we must deduce the bounds of $s(t),\dot{s}(t)$ to investigate the reachability of the sliding manifold.

It is easy to see that $E{\left|s(t)\right|}^{2}=E{\left|\mu_{1}(t)\right|}^{2}\leq \bar{\varepsilon }$ can be achieved within the time interval *T*, as for $\dot{s}(t)$, the following inequality can be obtained with reference to (6):

$$E{\left\|\dot{s}\right\|}^{2}=E{\left\|{\dot{\mu }}_{1}\right\|}^{2}\leq E{\left\|k_{1}{\left|\mu_{1}\right|}^{\frac{m-1}{m}}+k_{2}\mu_{1}+k_{3}\left|\mu_{2}\right|+\sum_{i=1}^{l}\eta_{i}\right\|}^{2}$$ (40)

The following inequality can be obtained by the Minkowski inequality

$$\begin{array}{l} \;{\left(E{\left\|k_{1}{\left|\mu_{1}\right|}^{\frac{m-1}{m}}+k_{2}\mu_{1}+k_{3}\left|\mu_{2}\right|+\sum_{i=1}^{l}\eta_{i}\right\|}^{2}\right)}^{1/2}\\ \leq {\left(E{\left\|k_{1}{\left|\mu_{1}\right|}^{\frac{m-1}{m}}\right\|}^{2}\right)}^{1/2}+{\left(E{\left\|k_{2}\mu_{1}\right\|}^{2}\right)}^{1/2}+{\left(E{\left\|k_{3}\left|\mu_{2}\right|\right\|}^{2}\right)}^{1/2}+\sum_{i=1}^{l}{\left(E{\left\|\eta_{i}\right\|}^{2}\right)}^{1/2}\\ =k_{1}{\left(E{\left|\mu_{1}\right|}^{\frac{2(m-1)}{m}}\right)}^{1/2}+k_{2}{\left(E{\left\|\mu_{1}\right\|}^{2}\right)}^{1/2}+k_{3}{\left(E{\left\|\mu_{2}\right\|}^{2}\right)}^{1/2}+l\sqrt{\bar{\varepsilon }}\\ \leq k_{1}{\left(E{\left|\mu_{1}\right|}^{\frac{2(m-1)}{m}}\right)}^{1/2}+(k_{2}+k_{3}+l)\sqrt{\bar{\varepsilon }} \end{array}$$ (41)

Then, by the Lyapunov inequality, we have

$${\left(E{\left|\mu_{1}\right|}^{\frac{2(m-1)}{m}}\right)}^{\frac{m}{2(m-1)}}\leq {\left(E{\left|\mu_{1}\right|}^{2}\right)}^{\frac{1}{2}}$$ (42)

It follows that

$$E{\left|\mu_{1}\right|}^{\frac{2(m-1)}{m}}\leq {\left(E{\left|\mu_{1}\right|}^{2}\right)}^{\frac{m-1}{m}}\leq {\bar{\varepsilon }}^{\frac{m-1}{m}}$$ (43)

Substituting (41), (43) into (40) yields

$$\sqrt{E{\left|\dot{s}\right|}^{2}}\leq k_{1}{\bar{\varepsilon }}^{\frac{m-1}{m}}+(k_{2}+k_{3}+l)\sqrt{\bar{\varepsilon }}$$ (44)

From the whole proving process, we notice that the parameter $\bar{\varepsilon }$ can be interpreted as the control precision index, so we can reasonably assume that $\bar{\varepsilon }$ is much less than 1 to meet the needs of engineering practice, and note that *m* > 2, we have

$$\sqrt{E{\left|\dot{s}\right|}^{2}}\leq (k_{1}+k_{2}+k_{3}+l)\sqrt{\bar{\varepsilon }}$$ (45)

So the following inequalities hold

$$\left\{ \begin{array}{l} E{\left|s\right|}^{2}\leq \bar{\varepsilon }\\ E{\left|\dot{s}\right|}^{2}\leq {\left(k_{1}+k_{2}+k_{3}+l\right)}^{2}\bar{\varepsilon } \end{array}\right.$$ (46)

Let $\varepsilon =[1+{\left(k_{1}+k_{2}+k_{3}+l\right)}^{2}]\bar{\varepsilon }$, by Definition 2, we can claim that the sliding manifold ***s*** = 0 is second-order finite-time mean-square practically reachable with respect to (*λ*,*ε*). So the proof is completed.

**Fig 1:**
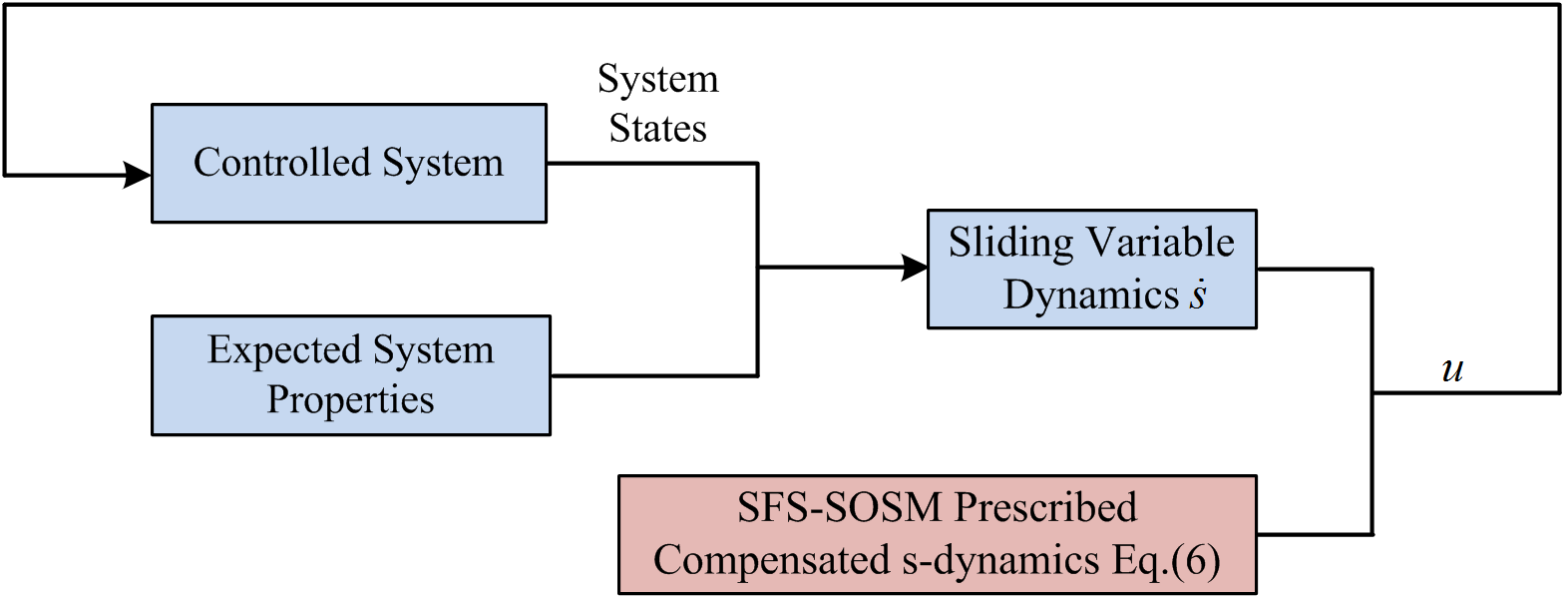
The block diagram of SFS-SOSM control design.

The control approach block-diagram of proposed SFS-SOSM method is shown in Fig 1.

The design process of the controller is: first, the sliding variable dynamics $\dot{s}(x)$, where ***x*** represent the system states, is obtained according to the expected system properties; Then the control law *u* is got by combining $\dot{s}(x)$ and the prescribed s-dynamics Eq(6); So the smooth control law *u* can steer the system state reach the desired value in finite time.

**Remark 2:** The proposed control law (10) is a SFS-SOSM control, which is smooth and can provides a finite time convergence $\left(E{\left|s\right|}^{2},E{\left|\dot{s}\right|}^{2})\to U_{\varepsilon }(0,0\right)$.

**Remark 3:** *ε* can be treated as the convergence precision. It can be seen from condition (i) that *ε* depends on the parameters of the colored noise and the designed parameters of the controller.

## Results

In this section, a second-order nonlinear stochastic system is taken into consideration to illustrate the necessity and effectiveness of the proposed control law.

Consider the following second-order SISO nonlinear stochastic system with colored noise

$$\left\{ \begin{array}{l} {\dot{x}}_{1}=x_{2}\\ {\dot{x}}_{2}=2x_{2}^{2}+u+d(t)+2\eta_{1}+2\eta_{2}+\eta_{3} \end{array}\right.$$ (47)

where

$$\begin{array}{l} {\dot{\eta }}_{1}=-2\eta_{1}+\zeta \\ {\dot{\eta }}_{2}=-4\eta_{2}+0.1\zeta \\ {\dot{\eta }}_{3}=-\eta_{3}+4\zeta \end{array}$$

and *ζ* is a zero-mean scalar Gaussian process with covariance 1. The initial state is (*x*_1_,*x*_2_) = (2,5).

In order to achieve finite time convergence, the following auxiliary integral sliding variable

$$s=0.5{\dot{x}}_{1}+1.5x_{1}+\int_{0}^{t}x_{1}$$ (48)

is introduced. This sliding surface can guarantee a finite-time convergence of the system state due to its nonlinear switching manifold characteristic.

The prescribed compensated *s-*dynamics providing finite-time mean-square convergence are selected in a format (6). In accordance with (10) the smooth control input is selected to be

$$\left\{ \begin{array}{l} \;u=2[-k_{1}{\left|\mu_{1}\right|}^{\frac{m-1}{m}}\mathrm{sgn}\left(\mu_{1}\right)-k_{2}\mu_{1}-k_{3}\left|\mu_{2}\right|\mathrm{sgn}(\mu_{1})-x_{2}^{2}-x_{1}-1.5x_{2}-0.5\hat{d}(t)]\\ {\dot{\mu }}_{2}=-k_{4}{\left|\mu_{1}\right|}^{\frac{m-2}{m}}\mathrm{sgn}\left(\mu_{2}\right)-k_{5}\mu_{2} \end{array}\right.$$ (49)

where the parameters are taken as *m* = 3, *k*_1_ = 20, *k*_2_ = 20, *k*_3_ = 1, *k*_4_ = 6, *k*_5_ = 6.

The effectiveness of the SFS-SOSM control is investigated by comparing the SFS-SOSM control with the smooth second-order sliding mode (SSOSM) control, which is designed to deal with deterministic systems. The SSOSM control is taken as [22]

$$\left\{ \begin{array}{l} u=2[-\alpha_{1}{\left|\mu_{1}\right|}^{\frac{m-1}{m}}\mathrm{sgn}\left(\mu_{1}\right)+\mu_{2}-x_{2}^{2}-x_{1}-1.5x_{2}-0.5\hat{d}(t)]\\ {\dot{\mu }}_{2}=-\alpha_{2}{\left|\mu_{1}\right|}^{\frac{m-2}{m}}\mathrm{sgn}\left(\mu_{1}\right) \end{array}\right.$$ (50)

where the parameters are taken as *m* = 3, *α*_1_ = 20, *α*_2_ = 6. In (49) and (50), $\hat{d}(t)$ is the estimation of uncertain function *d*(*t*) by means of observer presented in [22].

**Fig 2:**
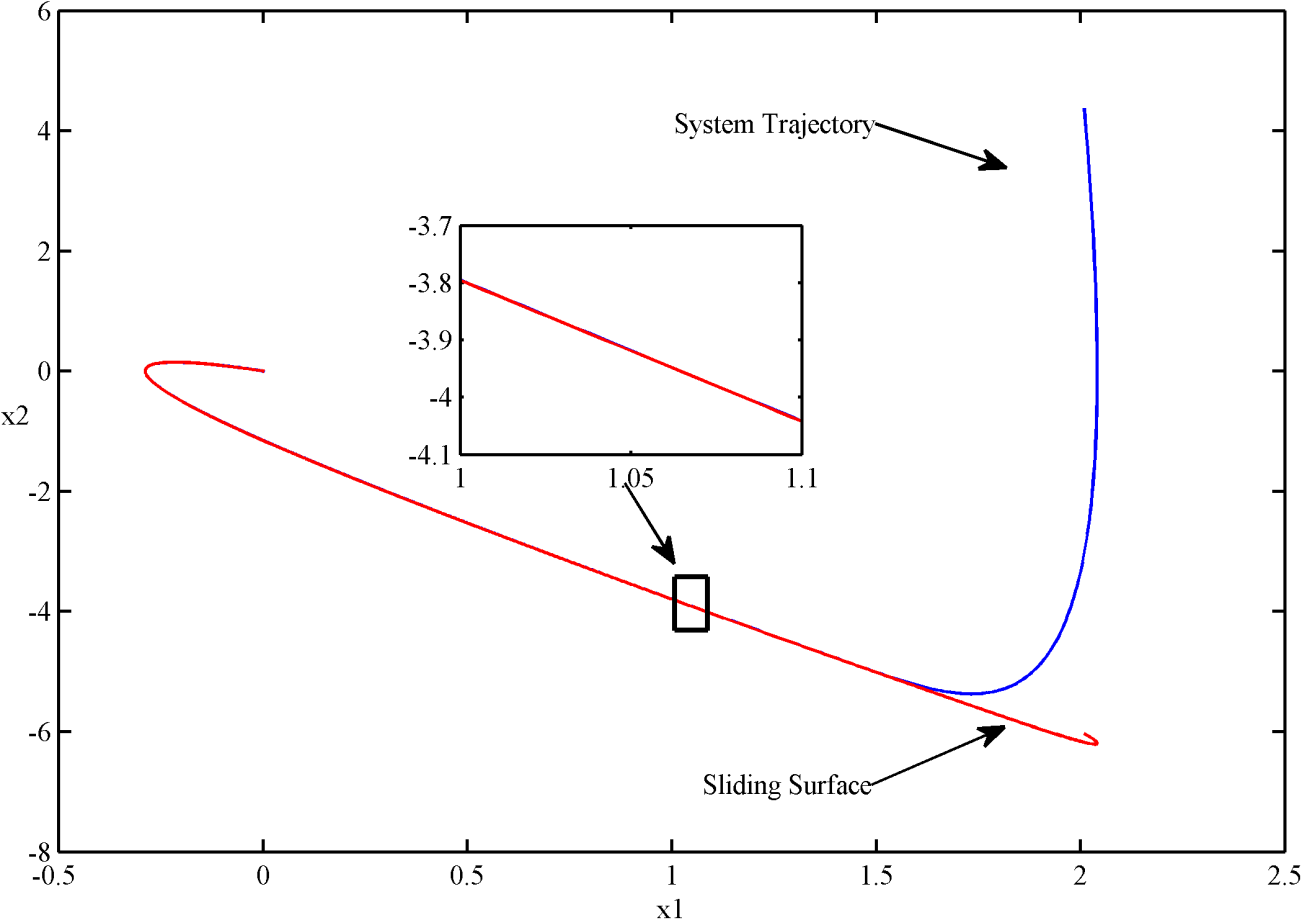
The phase plot of SFS-SOSM control.

**Fig 3:**
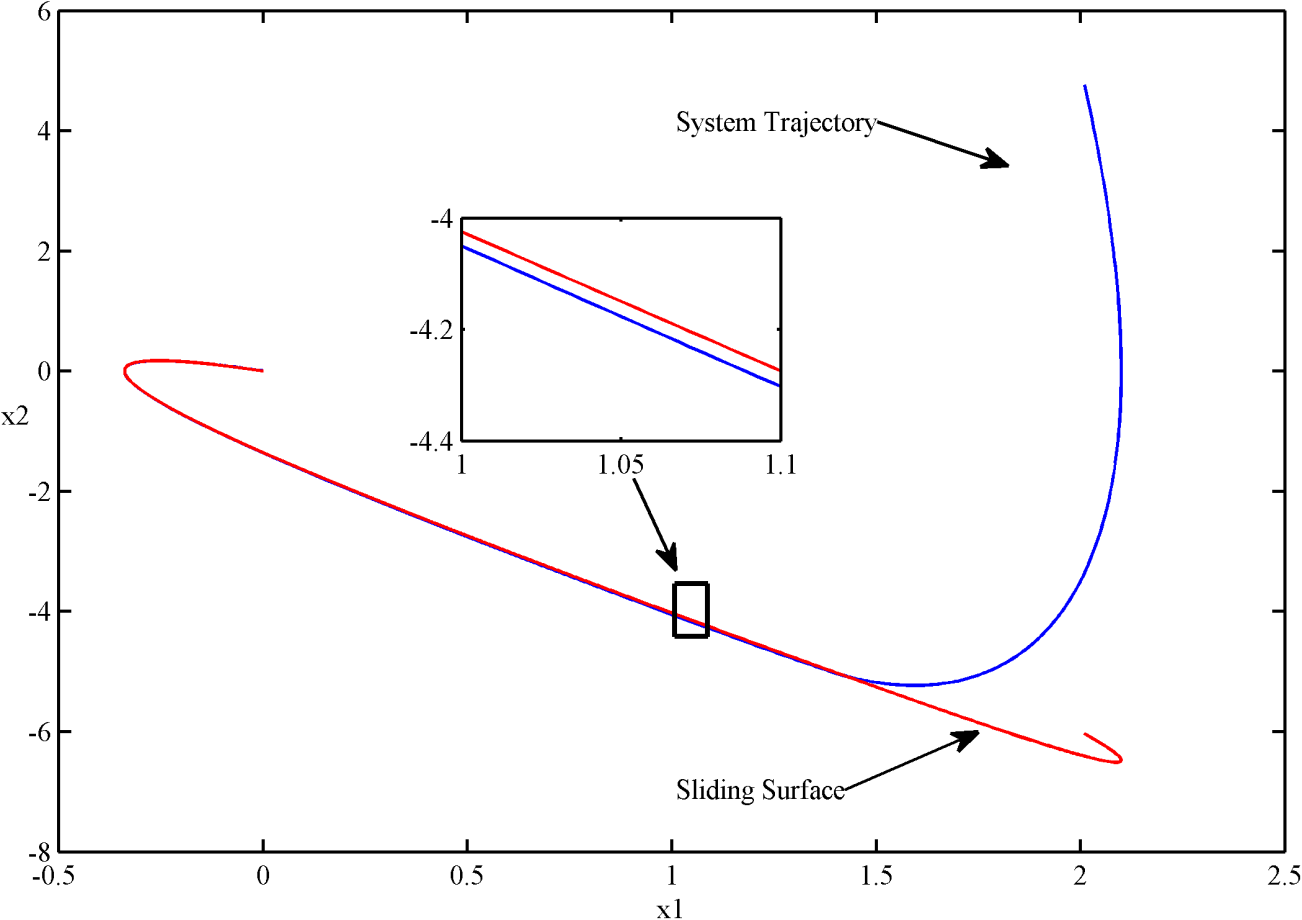
The phase plot of SSOSM control.

The phase plots of two kinds of control are shown in Figs 2 and 3.

It is obvious that both of the controllers can steer the system state from the initial position to the sliding surface, and then the sliding mode with state trajectories in this surface starts and thereafter remains on it. At the same time, the chattering of the sliding mode is eliminated in view of these figures.

From the partial enlargements of the Figs 2 and 3, we can see that the SFS-SOSM controller can steer the system trajectory closer to the sliding surface comparing with the SSOSM controller. This result demonstrates that the SFS-SOSM method can significantly improve the control precision, since the stochastic control techniques are employed to handle the noise. By contrast, the SSOSM controller adopts a more conservative control strategy, treating the stochastic noise as bounded uncertainties, which ensures the robustness at the cost of losing accuracy.

**Fig 4:**
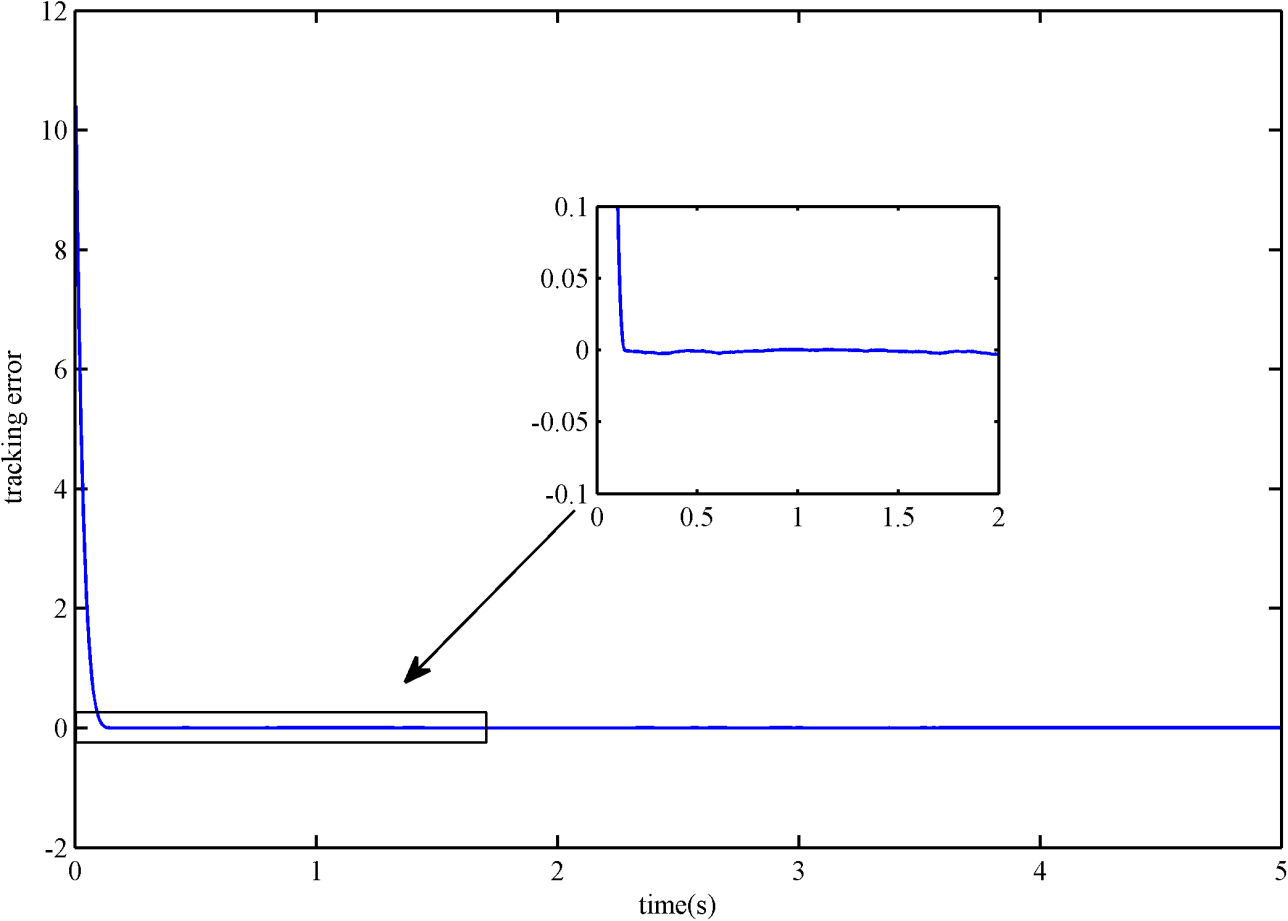
The tracking error of SFS-SOSM control.

**Fig 5:**
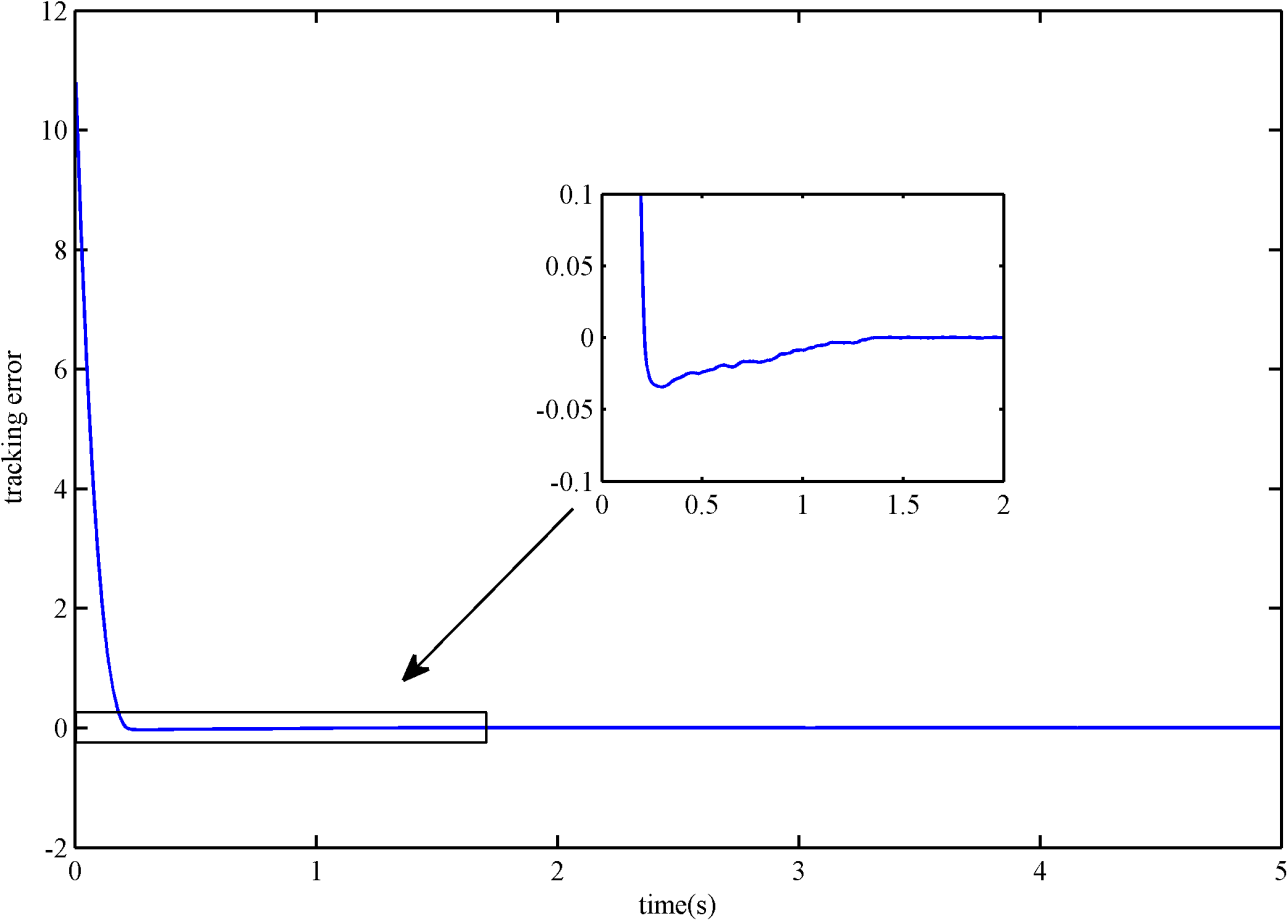
The tracking error of SSOSM control.

The trajectory tracking error is shown in Figs 4 and 5. It is obvious that the error convergence rate of SFS-SOSM is faster than SSOSM. The overshoot of SSOSM controller is larger than SFS-SOSM, which demonstrate that the SSOSM control is more conservative since it overestimates the bound of uncertainties.

**Fig 6:**
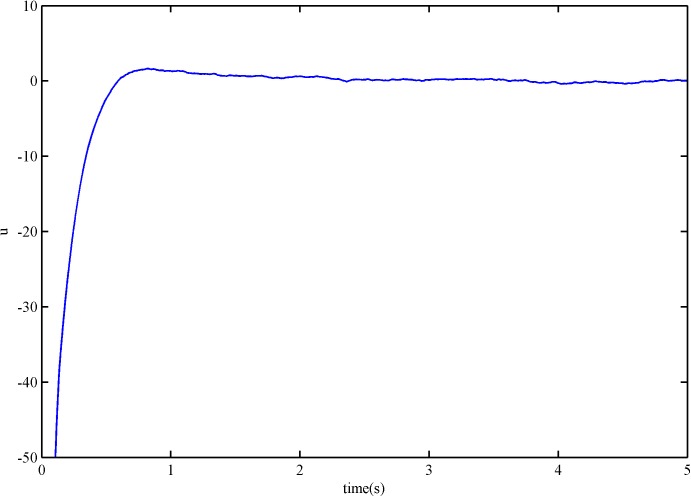
The control signal of SFS-SOSM control.

**Fig 7:**
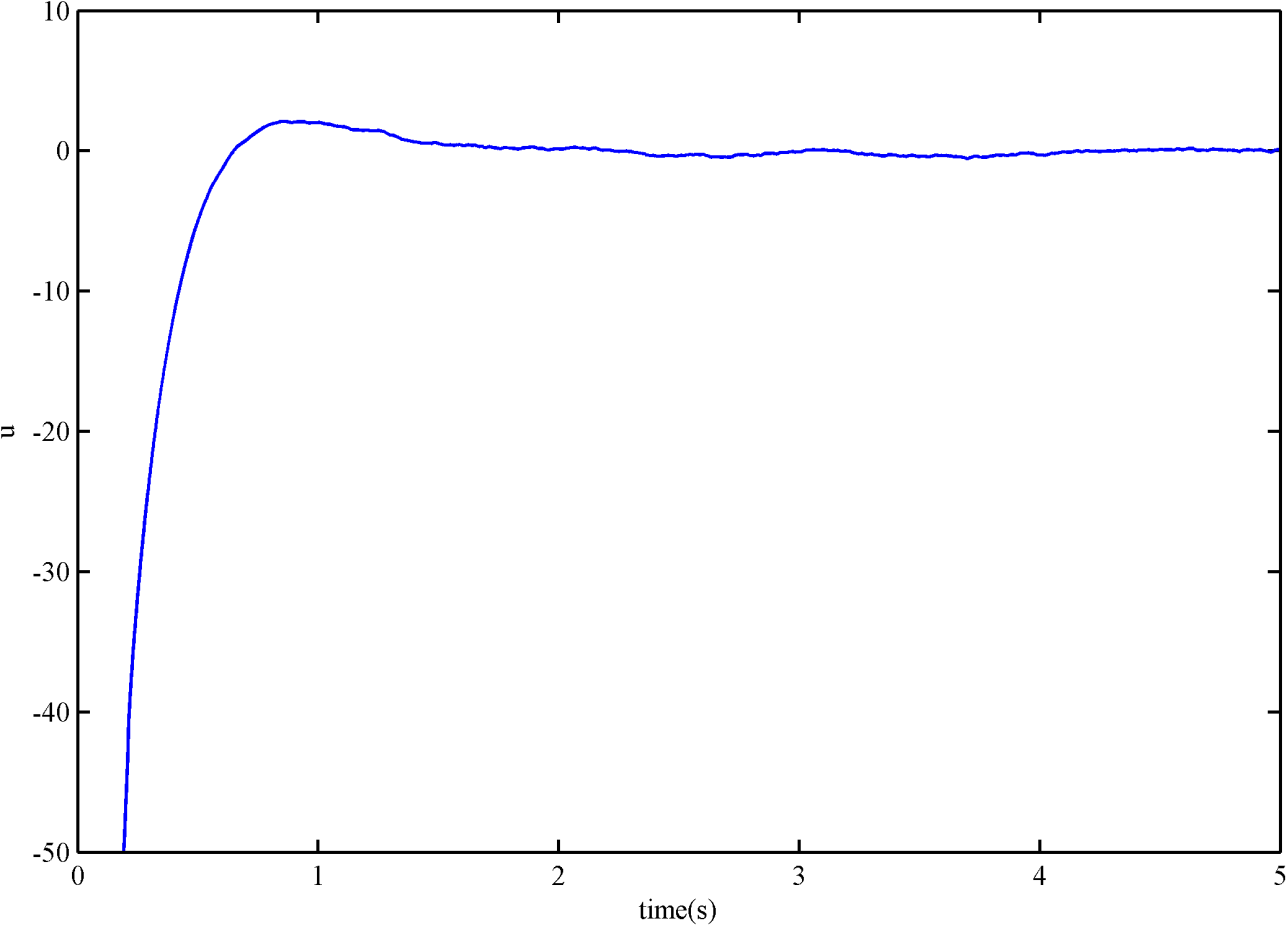
The control signal of SSOSM control.

The control signal of the SFS-SOSM controller and the SSOSM controller are presented in Figs 6 and 7. It is evident that neither of the controllers has high frequency switching benefited from the smooth controller design, but the overshoot of SSOSM controller is greater than SFS-SOSM.

**Fig 8:**
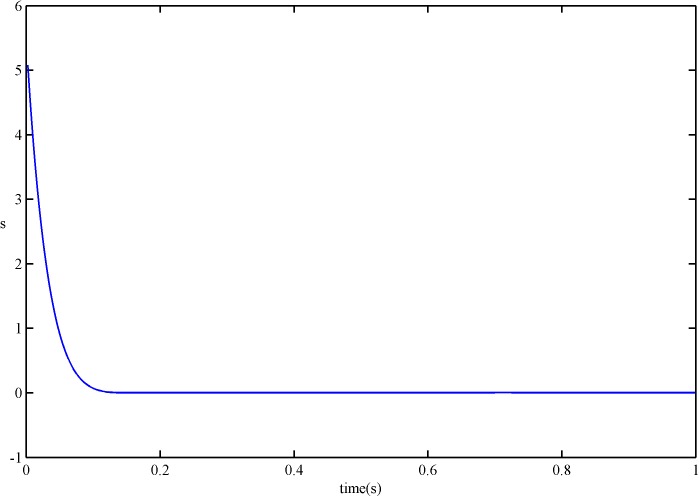
*s* of SFS-SOSM control.

**Fig 9:**
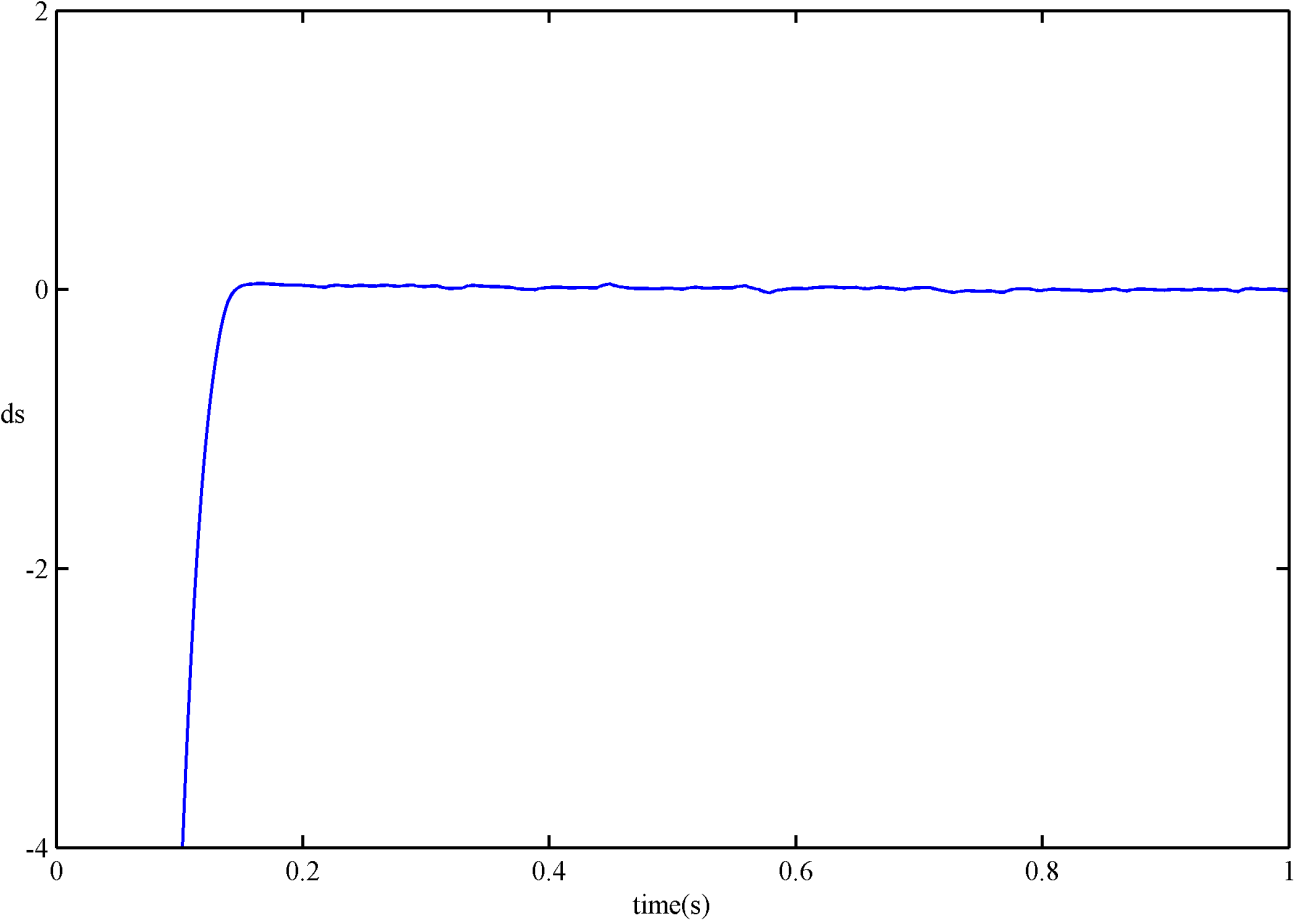
$\dot{s}$ of SFS-SOSM control.

Figs 8 and 9 show the simulated results of the sliding variable *s* and its derivative $\dot{s}$ under the SFS-SOSM control. From these figures, we can see that the proposed smooth control law can stabilize the sliding variable and its derivative at a sufficiently small neighborhood of zero in finite time, which means that the proposed control achieves the second-order sliding modes.

**Fig 10:**
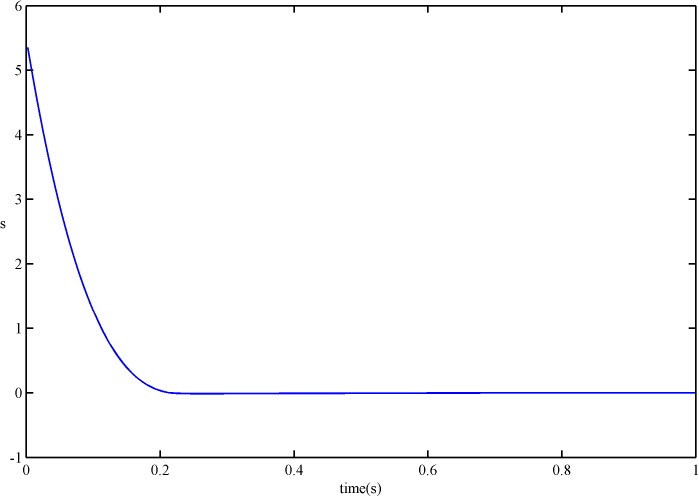
*s* of SSOSM control.

**Fig 11:**
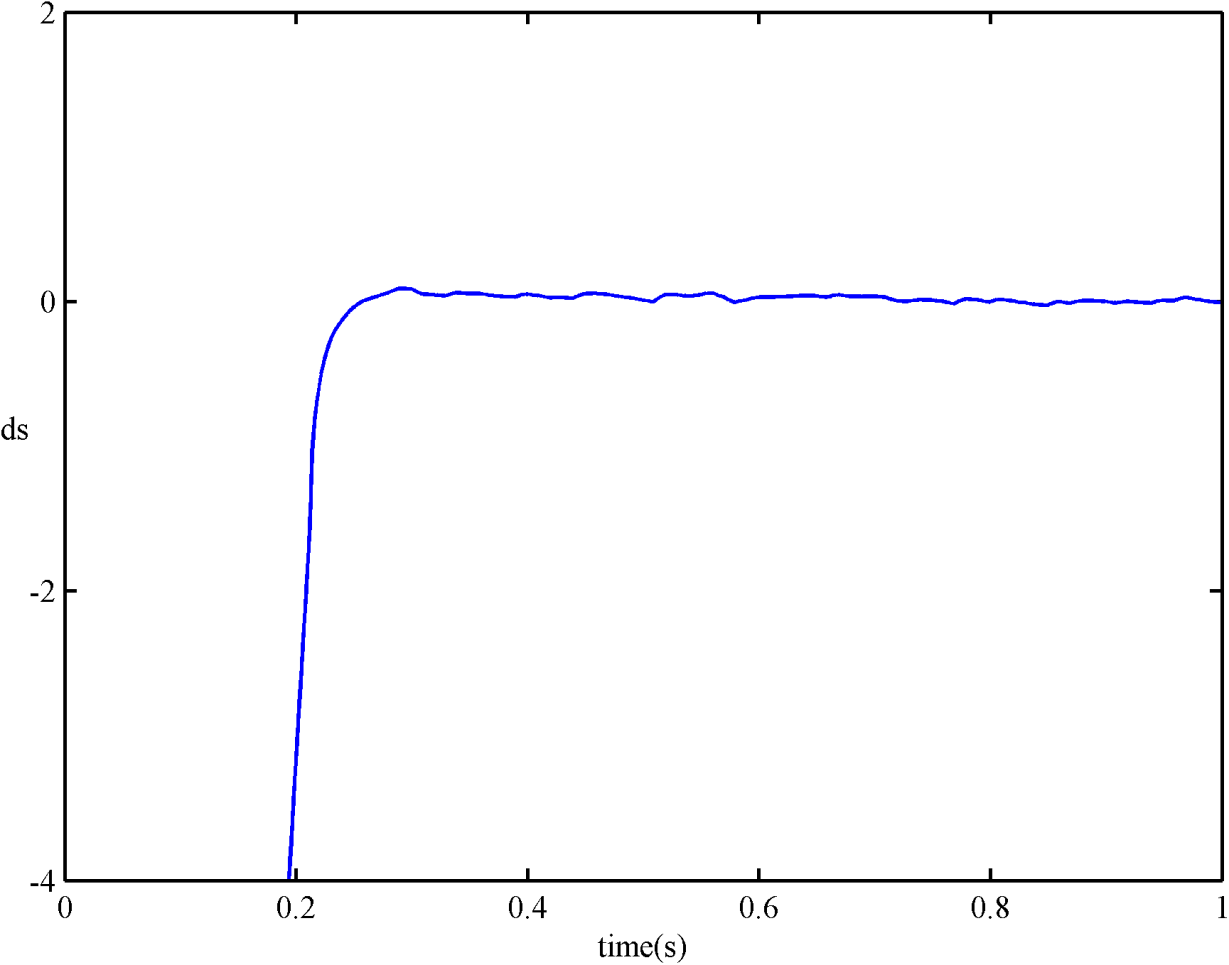
$\dot{s}$ of SSOSM control.

For comparison, results of *s* and $\dot{s}$ under the SSOSM control are presented in Figs 10 and 11. It is obvious that the convergence rate of *s* and $\dot{s}$ with the SFS-SOSM control is faster than the SSOSM control.

## Conclusions

In this paper, a SFS-SOSM controller for stochastic system with additive Ornstein-Uhlenbeck colored noise has been proposed. The time to achieve second-order reachability to the sliding manifold from initial system states has been proved to be finite. The new proposed sliding mode controller has the following advantages: first, it can eliminate the chattering associated with traditional sliding mode; second, it has no high frequency switching needed to be smooth at the price of losing robustness; third, it can achieve higher control accuracy since the stochastic technique is employed to design the controller instead of treating the noise as bounded uncertainty. Simulation results are presented to validate the analysis.

Future work includes optimizing the controller parameters to achieve better control performance and applying the proposed control to the practice engineering problems. We will also consider designing a more perfect disturbance observer to replace observer presented in [22] to improve the control precision.

## Supporting information

S1 Table The Simulation Data of SFS-SOSM Control. (XLSX)

S2 Table The Simulation Data of SSOSM Control. (XLSX)
